# Nursing Process in Human Milk Banks: technology development and validity

**DOI:** 10.1590/0034-7167-2025-0206

**Published:** 2026-07-27

**Authors:** Camila Trevisan Saldanha, Silvana dos Santos Zanotelli, Carla Argenta, Edlamar Kátia Adamy, Cassiana Mendes Bertoncello Fontes

**Affiliations:** I Universidade do Estado de Santa Catarina. Chapecó, Santa Catarina, Brazil; II Universidade Estadual Paulista, Faculdade de Medicina de Botucatu. São Paulo, São Paulo, Brazil

**Keywords:** Nursing Process, Technology, Nursing, Milk Banks, Breast Feeding., Proceso de Enfermería, Tecnología, Enfermería, Bancos de Leche Humana, Lactancia Materna.

## Abstract

**Objectives:**

to construct and validate a nursing assessment instrument and care matrix for implementing the Nursing Process in a Human Milk Bank.

**Methods:**

methodological research, carried out in two stages: evidence-based instrument construction using an integrative review, the Theory of Basic Human Needs, and the NANDA International, Nursing Outcomes Classification, and Nursing Interventions Classification taxonomies; and content validity by 12 experts using an electronic form. Data analysis was performed using the Content Validity Index and the Kappa Index.

**Results:**

the instrument consists of two parts: initial assessment; and a matrix with nursing diagnoses, outcomes, and interventions. The first part had a Content Validity Index and Kappa Index of 0.84 and 0.74, respectively. The second part had a Content Validity Index of 0.96 and a Kappa Index of 0.86.

**Conclusions:**

the technology proved valid in terms of content, with the potential to standardize and guide nursing practices.

## RESUMEN

**Objetivos:**

construir y validar un instrumento de valoración de enfermería y una matriz de cuidados para la implementación del Proceso de Enfermería en un Banco de Leche Humana.

**Métodos:**

investigación metodológica, realizada en dos etapas: construcción de un instrumento basado en la evidencia mediante una revisión integrativa, la Teoría de las Necesidades Humanas Básicas y las taxonomías de NANDA International, Nursing Outcomes Classification y Nursing Interventions Classification; y la validación de contenido por 12 expertos mediante un formulario electrónico. El análisis de datos se realizó utilizando el Índice de Validación de Contenido y el Índice Kappa.

**Resultados:**

el instrumento consta de dos partes: una evaluación inicial y una matriz con diagnósticos, resultados e intervenciones de enfermería. La primera parte tuvo un Índice de Validación de Contenido de 0,84 y un Índice Kappa de 0,74. La segunda parte tuvo un Índice de Validación de Contenido de 0,96 y un Índice Kappa de 0,86.

**Conclusiones:**

la tecnología demostró validez de contenido y potencial para estandarizar y orientar la práctica de enfermería.

## INTRODUCTION

The Nursing Process (NP) is a method that guides nurses’ critical thinking and clinical judgment, directing the nursing team towards the care of individuals, families, communities, and special groups^(1)^. It originated in the 1950s in the United States, and although the term NP was not used, the pioneer of nursing, Florence Nightingale, already highlighted the importance of nurses’ observation and judgment^(2)^.

In Brazil, NP implementation is currently regulated by Resolution 736/2024, which provides for its implementation in all socio-environmental contexts where nursing care occurs^(1)^. NP has become prominent in nurses’ professional practice, conferring autonomy and strengthening the professional category, as it instrumentalizes practice and documents the care provided; however, it is still underestimated by some professionals^(3)^.

Even if in a disorganized way and without standardized language, many nurses have been implementing NP in their practice through nursing consultation (NC), which must be organized and recorded according to the NP stages^(1)^. NP and NC are methodologies or instruments to be implemented in all contexts of nursing care for human beings.

In the context of a Human Milk Bank (HMB), characterized as a specialized service, several stages are necessary in the care process, such as collecting, processing, and distributing human milk safely and with high quality. Thus, it is a reference point in assisting pregnant women, postpartum women, and breastfeeding mothers, focusing on breastfeeding (BF) promotion, protection, and support^(4)^.

Concerning the importance of nursing care in BF, a recent study reflects the concern surrounding the topic in order to achieve better results in this practice, portraying nurses’ role as a determining factor in influencing mothers’ decisions to breastfeed^(5)^. In this regard, nurses’ role in HMBs should be guided by technical guidelines from the Brazilian National Health Regulatory Agency^(6)^ and organized by NP. Nurses’ clinical judgment derived from data collection guides the choice of nursing diagnoses (NDs), enabling the planning of actions directed to the specificities of identified phenomena^(7)^.

The regulations that guide nurses’ role in HMBs are comprehensive; however, they do not include a tool to guide NP implementation^(8)^. When an instrument is constructed and organized for a specific population, it allows for complete data collection, which corroborates the establishment of the nurse-patient bond. Thus, this instrument encompasses and guides the NP stages, such as defining NDs, planning, implementation, and nursing assessment^(9)^.

In this context regarding NP implementation in a HMB, the construction and validity of an instrument-type technology that includes nursing assessment, diagnoses, outcomes, and interventions is justified. For nursing assessment, it is important to consider the collection of objective and subjective information that ensures a comprehensive analysis of nursing mothers’ health conditions and biopsychospiritual needs^(10)^. Considering the relevance of NDs, outcomes, and interventions were based on internationally recognized taxonomies, such as NANDA International^(11)^, Nursing Outcomes Classification^(12)^ and Nursing Interventions Classification^(13)^(NNN). A technology that has a methodology for organizing care with a view to improving the quality and efficiency of care provided to the population enables the qualification of nurses’ performance and decision-making in work and care management and processes^(14)^.

Therefore, the proposed technology aims to support NP implementation in HMBs, ensuring qualified nursing care, supporting BF promotion, protection, and encouragement, and nursing record documentation.

## OBJECTIVES

To construct and validate a nursing assessment instrument and care matrix for NP implementation in HMBs.

## METHODS

### Ethical aspects

The study complied with the ethical precepts in force in Resolution 466/2012^(15)^ of the Brazilian National Health Council, and was approved by the *Universidade do Estado de Santa Catarina* Research Ethics Committee, under Opinion 6,775,536.

All procedures were carried out online after agreement and signing of the Informed Consent Form (ICF).

### Study design

This is methodological research, developed in two stages: construction of an instrument for NP implementation; and validity of its content^(16-18)^.

### Study setting

#### Setting 1

This research stems from a HMB’s needs in the state of Santa Catarina. HMB is part of a highly complex structure within a leading institution for maternal and child health, assisting a population of over one million inhabitants, with 310 inpatient beds and holding the title of Baby-Friendly Hospital since 1998.

#### Setting 2

The validity phase of the tool involved nurses with expertise in assisting BF mothers, working in HMBs in the state of Santa Catarina, and nurses who are members of *Rede de Pesquisa em Processo de Enfermagem* (RePPE). These nurses answered a questionnaire on Google Forms^®^ (online).

### Study stages

#### First stage: construction of an instrument for implementing the Nursing Process

The instrument content was defined based on documents from the Brazilian National Health Regulatory Agency and the Brazilian Network of Human Milk Banks, as well as the researchers’ experience from the perspective of Wanda Aguiar Horta’s Theory of Basic Human Needs. The instrument’s formatting followed standards available in the literature^(19,20)^.

The construction phase took place between July and October 2024, and the instrument had two parts: the first with content for nursing assessment, based on Wanda Aguiar Horta’s Theory of Basic Human Needs^(10)^, considering psychobiological, psychosocial and psychospiritual human needs, in the first stage of NP, and in Art. 2° of COFEN Resolution 736/2024^(1)^, which highlights that nursing assessment must be based on theoretical support, including objective and subjective data (anamnesis and physical examination).

The second part of the instrument includes the NNN matrix, developed in accordance with the NANDA-I NDs^(11)^, interventions and activities from the Nursing Interventions Classifications (NIC)^(13)^, and outcomes and indicators from the Nursing Outcomes Classifications (NOC)^(12)^ applicable to nursing phenomena in a HMB, to the likely problems to be recognized in nursing assessment, and to the establishment of NNN linkages.

To identify the NDs described in the matrix, the titles of NDs from the NANDA I version 2021-2023 taxonomy, were read, and diagnoses were pre-selected for reading the definition, defining characteristics, related factors, associated conditions, and at-risk population. After analysis by the researchers, 24 NDs were included in the matrix.

Subsequently, a search for nursing outcomes in the NOC was conducted using titles, followed by an analysis of the definition and indicators. Twenty-five NOC outcomes and indicators were selected, with the possibility of linking them to the 24 NDs. Similarly, in order to establish the link between nursing interventions and diagnoses, 25 intervention titles and their corresponding activities were listed.

A thorough search of NANDA-I, NIC, and NOC taxonomies resulted in the construction of 24 links between NDs, interventions, and outcomes, presented in the care matrix, the second part of the instrument for implementing NP in HMBs.

#### Second stage of the study: content validity

In this stage, the content validity of the instrument for NP implementation in HMB took place, from October to December 2024, with the participation of 12 specialist nurses who met at least two of the following four inclusion criteria: having clinical-assistance experience with breastfeeding mothers for at least six months; being a specialist (*lato sensu* and/or *stricto sensu*) in maternal and child health, BF and/or NP; having publications related to the topic; and being a member of the scientific society in the area^(16,17)^.

Specialist recruitment was done through purposive sampling. Contact was made via instant messaging application with nurses working in HMBs in the state of Santa Catarina and with members of RePPE, an entity that brings together researchers from higher education and health institutions in Brazil. One of the researchers involved participates in the state commission of HMBs, while another is part of the RePPE group. These connections were fundamental to facilitating access and contact with professionals.

Invitation letters were sent to experts outlining the research project’s purpose, the inclusion criteria, and requesting an expression of interest in participating. All participants who expressed interest and met pre-established inclusion criteria were accepted.

Those interested in participating were individually sent, via instant messaging application, the ICF, along with a link to access NP implementation instrument in an electronic form. The form was divided into sections: 1) ICF reading and acceptance; 2) specialist characterization; 3) instructions for completing the validity form for NP implementation instrument, organized in two parts (nursing assessment and care matrix).

Response options followed a Likert-type scale, which rates experts’ opinion from 1 to 4, where 1 (Inadequate), 2 (Partially adequate), 3 (Adequate), and 4 (Completely adequate). At the end of each question, there was an opportunity for comments.

### Data analysis

For data analysis, the Content Validity Index (CVI) was used, which is the proportion between valid judgments and the total number of assessments for each item. A CVI ≥ 0.80^(17)^ was accepted for both individual items and for the overall score.

Furthermore, the Kappa Index (K) was applied to measure the agreement among experts’ responses per item, considering the inclusion of an item when K ≥ 0.61 for both the items and the overall score.

## RESULTS

In the content validity phase, 12 specialists participated, all of whom were women, predominantly aged 36 to 45 years (55%). As for academic qualifications, the majority held a specialization degree (67%). As for their experience in assisting breastfeeding and nursing mothers, the majority (75%) had five or more years of experience. Concerning their experience in HMBs, 45% of participants had worked in this service for between one and five years, and 58% performed NP in their institutions, but not specifically in HMBs.

The part of the nursing assessment instrument that includes nursing information contains data on sociodemographic information, obstetric history, examination results, current BF status, breast assessment, vital sign recording, followed by basic human needs (psychobiological, psychosocial, and psycho-spiritual) based on the Theory of Basic Human Needs.

In the second part of the instrument, the matrix is presented with the links between NDs, interventions, and outcomes applicable in the context of HMBs, aligning with the issues identified in nursing assessment.

The final version of the instrument can be accessed in its entirety via link https://www.udesc.br/arquivos/ceo/id_cpmenu/4901/Instrumento_Camila_17575048243068_4901.pdf.

In the validity phase, experts answered a content validity form using a Likert scale (1-4), with the possibility of providing suggestions in the form of comments at the end of each question. Validity was divided into two chunks. The first chunk contained questions relevant to nursing assessment. The second chunk contained items related to the NNN matrix.

Table 1 presents the content validity data.

**Table 1:** Overall Content Validity Index and Kappa Index of items comprising the nursing assessment instrument, Chapecó, Santa Catarina, Brazil, 2025

Items	I	P*A*	A	TA	CVI	Kappa
Sociodemographic data	0	3	5	4	0.75	0.62
Obstetric history	0	2	6	4	0.83	0.71
Examination results	0	2	6	4	0.83	0.71
Current breastfeeding status	0	3	6	3	0.75	0.60
Breast examination	0	2	6	4	0.83	0.71
Vital signs record	1	1	7	3	0.83	0.75
Psychobiological needs (chunk 1)	0	2	8	2	0.83	0.71
Psychobiological needs (chunk 2)	0	1	9	2	0.91	0.86
Psychobiological needs (chunk 3)	0	1	9	2	0.91	0.86
Psychosocial needs (chunk 1)	0	2	8	2	0.83	0.75
Psychosocial needs (chunk 2)	0	2	8	2	0.83	0.71
Psychospiritual needs (chunk 3)	0	1	9	2	0.91	0.85
TOTAL Content Validity Index/Kappa					0.84	0.74

*I - Inadequate; PA - Partially adequate; A - Adequate; TA - Totally adequate; CVI - Content Validity Index.*

The overall validity index of the nursing assessment instrument was 0.84, confirmed by a K-score of 0.74, thus validating it in the first round.

However, the items corresponding to sociodemographic data and current BF conditions were not validated in the first round, as they reached a CVI of 0.75 and a K of 0.60. Thus, according to experts’ relevant suggestions, the two chunks of the instrument were revised by the researchers and adjusted, as shown in Chart 1.

**Chart 1:** Suggestions from experts after the first round of content validity of the instrument for implementing the Nursing Process in a Human Milk Bank, Chapecó, Santa Catarina, Brazil, 2025

N°	Suggestion	Action
**Sociodemographic data**		
**E3**	What would be the reason for sharing information about WhatsApp scams in relation to surveys?	Accepted, withdrawn.
**E3**	Will there be any data to identify the patient, such as a taxpayer identification number, national identity card number, or mother’s name?	Not accepted. This data was not included because it is admission data already included in the service user’s registration. Nursing assessment data was considered in this stage.
**E8**	Home collection would be for donors. So, perhaps the question should be asked if a patient is a donor/potential donor, because who would these processes be for? Any patient who is served by the Human Milk Bank or only donors?	Accepted, donor identification item included.
**E10**	A question could be included to address difficulties or ease of accessing the milk bank, accessibility, or reduced mobility.	If not addressed, mobility issues are covered under the category of psychobiological needs.
**Current breastfeeding conditions**		
**E3**	Include information about laser therapy (performs laser therapy).	Accepted. Information regarding laser therapy added.
**E3**	“Normal” is also a bad term to use. Normal for whom? What is normal production?	Accepted. Appropriate terminology was used to reflect clinical expectations.
**E10**	Identify any breastfeeding difficulties and specify those that are currently supported by scientific evidence in the literature, using closed-ended questions (as proposed in this questionnaire).	Accepted, with added items such as pain while breastfeeding, difficulty latching, difficulty positioning, interference from nipples/bottles/pacifiers, decreased milk flow, and increased milk flow.
**E11**	In the sentence “is breastfeeding”, add “breastfeeding supplemented”.	Included item: “Breastfeeding supplemented with: artificial formula; other types of milk; food”.

It is noteworthy that, even in the items validated in the first round, such as obstetric history, breast assessment, psychobiological needs (sexuality), and psycho-spiritual needs, the researchers accepted suggestions considered relevant to the instrument.

In the section on obstetric history, an item was included regarding planned pregnancy, participation in courses and/or guidance on BF/breast care/perinatal education during prenatal care.

In the breast assessment section, information was included regarding the history of breast diseases and family history of breast cancer. In the psychobiological needs section, under the item sexuality, a question was included about the use of contraceptive methods. In the psycho-spiritual needs section, questions were included regarding religious beliefs and the practice of actions to strengthen spirituality.

In the second part of the instrument validity form for implementing NP in HMBs, experts assessed the links between NDs, interventions, and outcomes, where the total CVI was 0.96, and the concordance index was K: 0.86, as shown in Table 2.

**Table 2:** Content validity of the NANDA International, Nursing Outcomes Classification, and Nursing Interventions Classification (second part of the instrument for implementing the Nursing Process in a Human Milk Bank) matrix, Chapecó, Santa Catarina, Brazil, 2025

Connection number - nursing diagnosis	I	PA	A	TA	CVI	Kappa
1 - Ineffective breastfeeding	0	1	9	2	0.92	0.84
2 - Interrupted breastfeeding	0	1	9	2	0.92	0.84
3 - Readiness for Enhanced breastfeeding	0	0	10	2	1	0.89
4 - Insufficient breast milk production	0	1	9	2	0.92	0.84
5 - Risk for nipple-areolar complex injury	0	1	9	2	0.92	0.84
6 - Impaired tissue integrity	0	0	10	2	1	0.89
7 - Imbalanced nutrition less than body requirements	0	0	10	2	1	0.89
8 - Risk for imbalanced fluid volume	0	0	10	2	1	0.89
9 - Risk for constipation	0	3	7	2	0.75	0.63
10 - Disturbed sleep pattern	0	1	9	2	0.92	0.84
11 - Risk for infection	0	0	10	2	1	0.89
12 - Ineffective childbearing process	0	0	10	2	1	0.89
13 - Acute pain	0	0	10	2	1	0.89
14 - Situational low self-esteem	0	0	10	2	1	0.89
15 - Readiness for enhanced parenting	0	0	10	2	1	0.89
16 - Interrupted family processes	0	0	10	2	1	0.89
17 - Anxiety	0	0	10	2	1	0.89
18 - Fear	0	0	10	2	1	0.89
19 - Risk for caregiver role strain	0	1	10	1	0.92	0.84
20 - Risk for impaired attachment	0	0	10	2	1	0.89
21 - Impaired comfort	0	1	9	2	0.92	0.84
22 - Disturbed body image	0	0	10	2	1	0.89
23 - Readiness for enhanced spiritual well-being	0	1	9	2	0.92	0.84
24 - Spiritual distress	0	1	9	2	0.92	0.89
Total Content Validity Index					0.96	0.86

*I - Inadequate; PA - Partially adequate; A - Adequate; TA - Totally adequate; CVI - Content Validity Index.*

In this validity stage, the only item that did not reach the CVI to be considered validated was link 9 referring to the “Risk for constipation” ND, which had a CVI of 0.75. Therefore, it was decided to exclude the item, considering the observations recorded by experts, who did not consider this ND relevant to the context of HMBs.

## DISCUSSION

The role of nursing professionals in promoting, protecting, and supporting BF takes place in various settings, and the use of technology has become particularly important. Educational technologies, followed by clinical technologies, are the most commonly used in communication with women during BF^(21)^. The technologies used in nursing care result from the articulation of scientific knowledge to produce material or non-material goods that support effective interventions in care practice^(22)^.

Nursing care in HMB, guided by NC, promotes autonomy, raising the quality of care provided by nurses^(23)^. NC should be based on the NP stages, which, in turn, is recognized as a strategy that aims to integrate best practices, based on scientific evidence, clinical knowledge and individual needs^(24)^.

Developing specific tools for data collection, investigation, organization, and analysis improves the efficiency and reliability of instruments^(18)^. In nursing, the validity of tools that guide practice represents an advancement in health technologies. Through these validated instruments, it is possible to offer more effective guidance in patient care, contributing to improved quality of care provided^(25)^.

The instrument for implementing NP in HMB reached the minimum CVI recommended in the literature^(17)^, which reinforces its relevance. The instrument developed for implementing NP in HMB guides NC, documenting nursing care for breastfeeding mothers.

Developing and validating instruments to support NP implementation in HMBs supports breast milk production promotion, protection, and support, allowing for comprehensive and organized data collection, facilitating the definition of diagnostic tests and, consequently, the implementation of subsequent NP stages.

In this regard, the use of nursing assessment instruments has been progressively increasing in nursing practice. Based on the Theory of Basic Human Needs principles, they contribute to holistic care focused on patients’ individuality^(26)^.

The use of a tool that standardizes care ensures assertiveness in nursing assistance. This was the finding of research in which an instrument for nursing care for preoperative pediatric patients was constructed and validated, based on the Theory of Basic Human Needs, encompassing NANDA-I, NIC, and NOC linkages, which achieved CVI scores of 0.90 and 1^(27)^.

The first part of the instrument for implementing NP in HMB, constructed in this work, includes sociodemographic data, important information for understanding the context in which BF occurs, since factors related to age, education level, and socioeconomic status influence BF practice. In a correlation between maternal income and newborn feeding (with breast milk + supplementation at all feedings), an income of three or more minimum wages negatively impacted exclusive BF practice in the maternity ward^(28)^.

Conversely, maternal education proves to be a determining factor for the success of BF. Therefore, by using a tool that guides nursing assessment, identifying the unique characteristics of each context, nurses direct their interventions through their clinical judgment^(29)^.

One of the points raised by experts for inclusion of information in the constructed instrument was regarding participation in perinatal education activities and guidance on BF during prenatal care. This suggestion is in line with protocol 19 of the Academy of Breastfeeding Medicine^(30)^, revised in 2024, which addresses the importance of promoting BF in the prenatal context.

The protocol emphasizes the importance of preparing pregnant women for BF, carried out by professionals during prenatal care, addressing the benefits of BF, dispelling misconceptions, promoting and directing pregnant women to BF support groups, as well as teaching practical skills such as latch and positioning. The protocol highlights the importance of partners/support network’s involvement throughout the process and ongoing follow-up after birth^(30)^.

However, a prospective cohort study with 300 postpartum women found that the provision of BF guidance during the pregnancy-postpartum period is below the recommended level, as approximately 50% of the studied population did not receive professional guidance on BF^(31)^.

The information from the subsequent sections of the nursing assessment instrument regarding obstetric history, current BF status, and breast assessment allows for the identification of obstacles and complications with a direct impact on nursing mothers’ health and BF outcome. Regarding pregnancy monitoring and the number of consultations, statistically significant differences were observed in the prevalence of BF at six months. Pregnant women who had five or more consultations maintained lactation at six months, unlike those who had fewer than five consultations^(29)^.

Reinforcing this premise, a study on BF self-efficacy identified that sociodemographic conditions, obstetric history, current pregnancy, and the postpartum period impact self-efficacy. Breastfeeding mothers should receive specialized support, and, according to this approach, it is fundamental that HMBs organize care through structured tools to provide quality assistance^(32)^.

Breast conditions, such as insufficient breast milk production, engorgement, fissures, fatigue, tiredness, and pain, are important factors that impact breastfeeding mothers’ physical and emotional health, requiring appropriate practices to minimize impacts and promote lactation maintenance^(33)^.

When examining factors that influence the interruption of exclusive BF, the authors^(34)^ identified that it occurred mainly due to lack of knowledge, as well as limiting beliefs, interference from friends and family, showing that one cannot consider only BF’s biological aspects, but must also consider psychological and sociocultural factors.

By examining modifiable factors one month postpartum related to exclusive BF three months postpartum, focusing on psychosocial variables such as breast complications, BF self-efficacy, stress levels, depressive symptoms, bonding, and family support, it was concluded that these factors influence exclusive BF continuation^(34)^.

In this way, a tool that considers the assessment of breastfeeding mothers in their uniqueness, taking into account their psychobiological, social, and spiritual conditions, based on the NNN matrix for defining NDs, outcomes, and interventions, contributes to the identification, planning, and early resolution of problems that may impact lactation maintenance.

### Study limitations

A limitation of this study was the difficulty in finding experts willing to participate and who met the proposed criteria. The fact that the study was conducted with experts from only one country may have limited the generalizability of results.

### Contributions to nursing, health, or public policy

Using a tool developed and validated specifically for breastfeeding mothers not only guides and standardizes procedures but also improves nursing assessment, nurses’ clinical judgment, the proper recording of interventions, and the outcomes achieved. Its innovative potential supports the promotion of maternal and child health wherever it is used, and its application can extend to other settings such as nursing offices, BF clinics, and Primary Health Care, as well as its use in NC.

## FINAL CONSIDERATIONS

The research highlighted the importance of developing a technology to improve care for breastfeeding mothers. NP, based on the NANDA-I, NIC, and NOC taxonomies, allows for the systematization of care, promoting a comprehensive, individualized, and scientifically sound approach.

With the development of assistive technology, we observed that nursing plays a central role in identifying breastfeeding mothers’ needs, proposing practical interventions, and assessing outcomes, contributing to favorable outcomes in maintaining lactation.

NP implementation fosters professional autonomy and standardization of practices. In the context of HMBs, it strengthens nurses’ commitment to promoting, protecting, and supporting BF.

The CVI achieved in this study demonstrates the reliability of the developed material as an instrument applicable in professional nursing practice, specifically in the context of HMB.

## Data Availability

The research data are available within the article.
