# Supplementary material for: Nursing Process in Human Milk Banks: technology development and validity
Source: Rev Bras Enferm. 2026 Jul 27;79:e20250206. doi: 10.1590/0034-7167-2025-0206 (PMC13411324; doi:10.1590/0034-7167-2025-0206)
Supplement: Translation

# Translation: Processo de Enfermagem em Banco de Leite Humano: construção e validação de tecnologia

**DOI:** 10.1590/0034-7167-2025-0206pt

**Authors:** Camila Trevisan Saldanha, Silvana dos Santos Zanotelli, Carla Argenta, Edlamar Kátia Adamy, Cassiana Mendes Bertoncello Fontes

**Affiliations:** I Universidade do Estado de Santa Catarina.Chapecó, Santa Catarina, Brasil; II Universidade Estadual Paulista, Faculdade de Medicina de Botucatu. São Paulo, São Paulo, Brasil

**Keywords:** Processo de Enfermagem, Tecnologia, Enfermagem, Bancos de Leite Humano, Aleitamento Materno.

## Abstract

**Objetivos:**

construir e validar instrumento de avaliação de enfermagem e matriz assistencial para implementação do Processo de Enfermagem em Banco de Leite Humano.

**Métodos:**

pesquisa metodológica, realizada em duas etapas: construção de instrumento baseado em evidências de revisão integrativa, Teoria das Necessidades Humanas Básicas, e taxonomias *NANDA International, Nursing Outcomes Classification* e *Nursing Interventions Classification*; e validação de conteúdo por 12 especialistas utilizando formulário eletrônico. A análise de dados se deu pelo Índice de Validação de Conteúdo e Índice Kappa.

**Resultados:**

o instrumento é constituído de duas partes: avaliação inicial; e matriz com diagnósticos, resultados e intervenções de enfermagem. A primeira parte teve Índice de Validação de Conteúdo 0,84 e Índice Kappa de 0,74. A segunda parte teve Índice de Validação de Conteúdo de 0,96 e Índice Kappa de 0,86.

**Conclusões:**

a tecnologia se mostrou válida quanto ao conteúdo, como potencial para padronizar e orientar as práticas do enfermeiro.

## INTRODUÇÃO

O Processo de Enfermagem (PE) é um método que orienta o pensamento crítico e o julgamento clínico do enfermeiro, direcionando a equipe de enfermagem para o cuidado à pessoa, família, coletividade e grupos especiais^(1)^. Originu-se na década de 50 nos Estados Unidos, e embora o termo PE não fosse utilizado, a precursora da enfermagem, Florence Nightingale, já destacava a importância da observação e julgamento do enfermeiro^(2)^.

No Brasil, atualmente, a implementação do PE é regulamentado pela Resolução nº 736/2024, que dispõe sobre a sua implementação em todo contexto socioambiental em que ocorre o cuidado de enfermagem^(1)^. O PE tem se destacado na prática profissional do enfermeiro, conferindo autonomia, e fortalecimento da categoria profissional, pois instrumentaliza a prática e documenta a assistência prestada; no entanto, ainda é subestimado por alguns profissionais^(3)^.

Mesmo que de forma desordenada e sem uma linguagem padronizada, muitos enfermeiros vêm executando o PE na sua prática por meio da consulta de enfermagem (CE), que deve ser organizada e registrada de acordo com as etapas do PE^(1)^. O PE e a CE são metodologias ou instrumentos a serem implementados em todos os contextos da assistência de enfermagem ao ser humano.

No contexto do Banco de Leite Humano (BLH), caracterizado com serviço especializado, são necessárias várias etapas no cuidado, como coletar, processar e distribuir leite humano com segurança e qualidade. Assim, é referência na assistência a gestantes, puérperas e nutrizes, com foco na promoção, proteção e apoio ao aleitamento materno (AM)^(4)^.

Acerca da importância da assistência de enfermagem ao AM, estudo recente reflete a inquietude do tema para alcançar melhores resultados dessa prática, retratando a atuação do enfermeiro como fator determinante para influenciar a decisão das mães de amamentar^(5)^. Neste sentido, a atuação do enfermeiro em BLH deve ser orientada por diretrizes técnicas da Agência Nacional de Vigilância Sanitária^(6)^ e organizada pelo PE. O julgamento clínico do enfermeiro advindo da coleta de dados direciona a escolha dos diagnósticos de enfermagem (DEs), possibilitando o planejamento das ações direcionadas às especificidades dos fenômenos identificados^(7)^.

As normativas que direcionam a atuação do enfermeiro nos BLHs são abrangentes, no entanto não contemplam uma ferramenta que direcione a implementação do PE^(8)^. Quando um instrumento é construído e organizado para uma determinada população, permite-se uma coleta de dados completa, que corrobora o estabelecimento do vínculo enfermeio-paciente. Assim, esse instrumento contempla e direciona as etapas do PE, como definição dos DEs, planejamento, implementação e avaliação de enfermagem^(9)^.

Nesse contexto sobre a implementação do PE em um BLH, justificam-se a construção e validação de tecnologia do tipo instrumento que contemple a avaliação, diagnósticos, resultados e intervenções de enfermagem. Para avaliação de enfermagem, é importante considerar a coleta de informações objetivas e subjetivas que garanta uma análise abrangente das condições de saúde e necessidades biopsicoespirituais^(10)^ da nutriz. Considerando a relevância dos DEs, resultados e intervenções se basearam nas taxonomias reconhecidas internacionalmente, como NANDA *International* ^(11)^, *Nursing Outcomes Classification* ^(12)^ e *Nursing Interventions Classification* ^(13)^ (NNN). Uma tecnologia que possua uma metodologia de organização da assistência com vistas a aprimorar a qualidade e a eficiência do atendimento prestado à população possibilita a qualificação da atuação do enfermeiro e a tomada de decisão na gestão e nos processos de trabalho e do cuidado^(14)^.

Destarte, a tecnologia proposta visa subsidiar a implementação do PE em BLH, assegurando um cuidado de enfermagem qualificado, corroborando a promoção, proteção e incentivo ao AM, e a documentação dos registros de enfermagem.

## OBJETIVOS

Construir e validar instrumento de avaliação de enfermagem e matriz assistencial para implementação do PE em BLH.

## MÉTODOS

### Aspectos éticos

O estudo cumpriu os preceitos éticos vigentes na Resolução n^o^ 466/2012^(15)^ do Conselho Nacional de Saúde, e foi aprovado pelo Comitê de Ética em Pesquisa com Seres Humanos da Universidade do Estado de Santa Catarina, sob Parecer n° 6.775.536.

Todos os procedimentos foram realizados de forma *online* após concordância e assinatura do Termo de Consentimento Livre e Esclarecido (TCLE).

### Tipo de estudo

Pesquisa metodológica, desenvolvida em duas etapas: construção de instrumento para implementação do PE; e validação de seu conteúdo^(16-18)^.

### Cenário do estudo

#### Cenário 1

Trata-se de pesquisa proveniente das necessidades de um BLH do estado de Santa Catatina. O BLH faz parte de uma estrutura de alta complexidade, em uma instituição de referência para saúde materno-infantil, atendendo uma população de mais de um milhão de habitantes, dispondo de 310 leitos de internação e detentor de título de Hospital Amigo da Criança desde 1998.

#### Cenário 2

Participaram da etapa de validação da ferramenta enfermeiros com *expertise* na assistência à nutriz, atuantes em BLHs no estado de Santa Catarina e enfermeiros membros da Rede de Pesquisa em Processo de Enfermagem (RePPE). Esses responderam a um questionário no Google Forms^®^ (*online*).

### Etapas do estudo

#### Primeira etapa: construção de instrumento para implementação do Processo de Enfermagem

A definição do conteúdo do instrumento se deu a partir de documentos da Agência Nacional de Vigilância Sanitária e Rede Brasileira de Bancos de Leite Humano, além da experiência das pesquisadoras sob a ótica da Teoria das Necessidades Humanas Básicas de Wanda Aguiar Horta. A formatação do instrumento seguiu padrões disponíveis na literatura^(19,20)^.

A etapa de construção ocorreu no período de julho a outubro de 2024, e o instrumento contou com duas partes: a primeira com conteúdo para avaliação de enfermagem, pautada na Teoria das Necessidades Humanas Básicas de Wanda Aguiar Horta^(10)^, considerando as necessidades humanas psicobiológicas, psicossociais e psicoespirituais, na primeira etapa do PE, e no Art. 2° da Resolução COFEN nº 736/2024^(1)^, que destaca que a avaliação de enfermagem deve estar fundamentada em suporte teórico, contemplando dados objetivos e subjetivos (anamnese e exame físico).

A segunda parte do instrumento contempla a matriz NNN, elaborada de acordo com os DEs da NANDA-I^(11)^, intervenções e atividades da *Nursing Interventions Classifications* (NIC)^(13)^ e resultados e indicadores da *Nursing Outcomes Classifications* (NOC)^(12)^ aplicáveis aos fenômenos de enfermagem de um BLH, aos prováveis problemas a serem reconhecidos na avaliação de enfermagem, e ao estabelecimento das ligações NNN.

Para identificar os DEs descritos na matriz, foram realizadas a leitura dos títulos de DE da taxonomia NANDA I, versão 2021-2023, e pré-seleção de diagnósticos para leitura da definição, características definidoras, fatores relacionados, condições associadas e população de risco. Após análise das pesquisadoras, 24 DEs foram incluídos na matriz.

Na sequência, realizou-se a busca dos resultados de enfermagem na NOC a partir dos títulos, com posterior análise da definição e indicadores. Foram selecionados 25 resultados NOC e indicadores, com possibilidade de ligação com os 24 DEs. Da mesma forma, com vistas a estabelecer a ligação das intervenções de enfermagem com os diagnósticos, elencara-se 25 títulos de intervenções e as atividades correspondentes.

A busca criteriosa nas taxonomias NANDA-I, NIC e NOC resultou na construção de 24 ligações entre diagnósticos, intervenções e resultados de enfermagem, apresentadas na matriz assistencial, segunda parte do instrumento para implementação do PE em BLH.

#### Segunda etapa do estudo: validação de conteúdo

Nesta etapa, ocorreu a validação de conteúdo do instrumento para implementação do PE em BLH, no período de outubro a dezembro de 2024, com a participação de 12 enfermeiros especialistas que contemplaram, no mínimo dois, dos quatro critérios de inclusão a seguir: ter experiência clínico-assistencial com nutrizes há pelo menos seis meses; ser especialista *lato sensu* e/ou *stricto sensu* em saúde materno-infantil, AM e/ou PE; possuir publicações relacionadas à temática; e ser membro da sociedade científica da área^(16,17)^.

O recrutamento dos especialistas se deu por amostragem intencional. Foi realizado contato por meio de aplicativo de mensagens instantâneas com enfermeiros atuantes em BLH do estado de Santa Catarina e com membros da RePPE, entidade que reúne pesquisadores de instituições de ensino superior e saúde do Brasil. Uma das pesquisadoras envolvidas participa da comissão estadual dos BLH, enquanto outra integra o grupo da RePPE. Essas conexões foram fundamentais para facilitar o acesso e o contato com os profissionais.

Foram enviadas cartas convite aos especialistas contendo a intenção do projeto de pesquisa, os critérios de inclusão e solicitação de manifestação de interesse na participação. Todos os participantes que manifestaram interesse e contemplavam os critérios de inclusão pré-estabelecidos.

Aos interessados em participar, foram encaminhados, individualmente, por meio de aplicativo de mensagens instantâneas, o TCLE, acompanhado do *link* de acesso ao instrumento de implementação do PE, em formulário eletrônico. O formulário foi dividido em sessões: l) leitura e aceite do TCLE; 2) caracterização dos especialistas; 3) instruções para preenchimento do formulário de validação do instrumento para implementação do PE, organizado em duas partes (avaliação de enfermagem e matriz assistencial).

As opções de respostas seguiram uma escala tipo Likert, que pontua de 1 a 4 a opinião dos especialistas, sendo 1 (Inadequado), 2 (Parcialmente adequado), 3 (Adequado) e 4 (Totalmente adequado). Ao final de cada questão, houve possibilidade para comentários.

### Análise dos dados

Para análise de dados, foi utilizado o cálculo do Índice de Validade de Conteúdo (IVC), que é a proporção entre os julgamentos válidos e o total de avaliações de cada item. Aceitou-se o IVC ≥ 0,80^(17)^ tanto para os itens quanto para o geral.

Ainda, aplicou-se o Índice Kappa (K) para medir a concordância entre as respostas dos especialistas por item, considerando-se a inclusão do item quando K ≥ 0,61 tanto para os itens quanto para o geral.

## RESULTADOS

Na etapa de validação de conteúdo, participaram 12 especialistas, sendo todas mulheres, com predomínio da faixa etária de 36 a 45 anos (55%). Em relação à titulação acadêmica, a maioria possuía grau de especialização (67%). Quanto ao tempo de atuação na assistência ao AM e às nutrizes, a maioria (75%) possuía cinco anos ou mais de atuação. Quanto ao tempo de atuação em BLH, 45% dos participantes atuavam nesse serviço entre um e cinco anos, e 58% realizavam o PE nas instituições de atuação, contudo não especificamente em BLH.

A parte do instrumento para implementação do PE que contempla avaliação de enfermagem possui informações referentes a dados sociodemográficos, antecedentes obstétricos, resultados de exames, condições referentes à amamentação atual, avaliação das mamas, registro dos sinais vitais, seguido das necessidades humanas básicas psicobiológicas, psicossociais e psicoespirituais fundamentas na Teoria das Necessidades Humanas Básicas.

Na segunda parte do instrumento, a matriz é apresentada com as ligações de diagnósticos, intervenções e resultados de enfermagem aplicáveis no contexto dos BLHs, aliando-se às questões identificadas na avaliação de enfermagem.

A versão final do instrumento poderá ser acessada na integra por meio de acesso ao link https://www.udesc.br/arquivos/ceo/id_cpmenu/4901/Instrumento_Camila_17575048243068_4901.pdf.

Na etapa de validação, os especialistas responderam a um formulário de validação do conteúdo, por meio da escala Likert (1-4), com possibilidade de sugestões no formato de comentários ao final de cada questão. A validação foi dividida em dois blocos. No primeiro bloco, estavam as questões pertinentes à avaliação de enfermagem. No segundo bloco, estavam os itens relacionados À matriz NNN.

Na Tabela 1, são apresentados os dados da validação de conteúdo.

**Quadro 1:** Sugestões dos especialistas após primeira rodada de validação de conteúdo do instrumento para implementação do Processo de Enfermagem em Banco de Leite Humano, Chapecó, Santa Catarina, Brasil, 2025

N°	Sugestão	Ação
**Dados sociodemográficos**		
**E3**	Qual motivo seria a informação sobre golpes de *WhatsApp* em relação a pesquisas?	Acatado, retirado.
**E3**	Não terá dados para identificação da paciente, como cadastro de pessoa física e registro geral, nome da mãe?	Não acatado. Tais dados não foram incluídos pois tratam-se de dados de admissão já contemplados no cadastro da usuária do serviço. Foram considerados nesta etapa dados de avaliação de enfermagem.
**E8**	Coleta domiciliar seria para doadoras. Então, talvez deveria ter a pergunta se a paciente é doadora/possível doadora, pois esses processos seriam para qual público? Qualquer paciente que for atendida ao Banco de Leite Humano ou apenas as doadoras?	Acatado, incluído item de identificação da doadora.
**E10**	Poderia ser incluída alguma pergunta ou para selecionar dificuldade ou facilidade para frequentar o banco de leite, acessibilidade ou mobilidade reduzida para tal.	Não acatado, questões de mobilidade estão contempladas no bloco de necessidades psicobiológicas.
**Condições da amamentação atual**		
**E3**	Incluir informação sobre laserterapia (faz laserterapia).	Acatado. Acrescentada informação referente à laserterapia.
**E3**	Normal também é um termo ruim de ser utilizado. Normal para quem? O que é produção normal?	Acatado. Utilizada terminologia adequada para expectativa clínica.
**E10**	Dificuldades identificadas para amamentação e especificar aquelas que são atualmente respaldadas por evidências científicas na literatura, utilizando perguntas fechadas (como está proposto nesse questionário).	Acatado, acrescentado itens como dor para amamentar, dificuldade na pega, dificuldade no posicionamento, interferência de bicos/mamadeira/chupeta, fluxo de leite diminuído e fluxo de leite aumentado.
**E11**	Em “está amamentando”, acrescentar “aleitamento materno complementado.	Incluído item “Aleitamento materno complementado: com fórmula artificial; outros tipos de leite; alimentos”.

**Tabela 1:** Índice de Validação de Conteúdo global e Índice Kappa dos itens que compõem o instrumento para avaliação de enfermagem, Chapecó, Santa Catarina, Brasil, 2025

Itens	I	P*A*	A	TA	IVC	Kappa
Dados sociodemográficos	0	3	5	4	0,75	0,62
Antecedentes obstétricos	0	2	6	4	0,83	0,71
Resultados de exames	0	2	6	4	0,83	0,71
Condições da amamentação atual	0	3	6	3	0,75	0,60
Avaliação das mamas	0	2	6	4	0,83	0,71
Registro dos sinais vitais	1	1	7	3	0,83	0,75
Necessidades psicobiológicas (bloco 1)	0	2	8	2	0,83	0,71
Necessidades psicobiológicas (bloco 2)	0	1	9	2	0,91	0,86
Necessidades psicobiológicas (bloco 3)	0	1	9	2	0,91	0,86
Necessidades psicossociais (bloco 1)	0	2	8	2	0,83	0,75
Necessidades psicossociais (bloco 2)	0	2	8	2	0,83	0,71
Necessidades psicoespirituais (bloco 3)	0	1	9	2	0,91	0,85
Índice de Validade de Conteúdo/Kappa TOTAL					0,84	0,74

*I - Inadequado; PA - Parcialmente adequado; A - Adequado; TA - Totalmente adequado; IVC - Índice de Validade de Conteúdo.*

O índice geral de validação do instrumento para avaliação de enfermagem foi de 0,84, confirmado pelo K de 0,74, sendo validado na primeira rodada.

No entanto, os itens correspondentes aos dados sociodemográficos e às condições da amamentação atual não foram validados na primeira rodada, pois atingiram um IVC de 0,75 e K de 0,60. Assim, de acordo com as sugestões pertinentes dos especialistas, os dois blocos do instrumento foram revisados pelas pesquisadoras e adequados, conforme Quadro 1.

Destaca-se que, mesmo nos itens validados na primeira rodada, como antecedentes obstétricos, avaliação das mamas, necessidades psicobiológicas (sexualidade) e necessidades psicoespirituais, as pesquisadoras acataram sugestões consideradas pertinentes ao instrumento.

No bloco de antecedentes obstétricos, foi incluído item referente à gestação planejada, à participação em cursos e/ou às orientações sobre AM/cuidados com as mamas/educação perinatal durante o pré-natal.

No bloco de avaliação das mamas, foram incluídas informações sobre o histórico de doenças mamárias e histórico familiar de câncer de mama. No bloco das necessidades psicobiológicas, item sexualidade, foi incluída a pergunta sobre uso de método contraceptivo. Nas necessidades psicoespirituais, foram incluídos questionamentos referentes à crença religiosa e à prática de ações para fortalecer a espiritualidade.

Na segunda parte do formulário de validação do instrumento para implementação do PE em BLH, os especialistas avaliaram as ligações entre diagnósticos, intervenções e resultados de enfermagem, em que o IVC total foi de 0,96, e o índice de concordância foi K: 0,86, conforme Tabela 2.

**Tabela 2:** Validação de conteúdo da matriz *NANDA International, Nursing Outcomes Classification* e *Nursing Interventions Classification* (segunda parte do instrumento para implementação do Processo de Enfermagem em Banco de Leite Humano), Chapecó, Santa Catarina, Brasil, 2025

Número da ligação - diagnóstico de enfermagem	I	PA	A	TA	IVC	Kappa
1 - Amamentação ineficaz	0	1	9	2	0,92	0,84
2 - Amamentação interrompida	0	1	9	2	0,92	0,84
3 - Disposição para amamentação melhorada	0	0	10	2	1	0,89
4 - Produção insuficiente de leite materno	0	1	9	2	0,92	0,84
5 - Risco de lesão no complexo aréolo mamilar	0	1	9	2	0,92	0,84
6 - Integridade tissular prejudicada	0	0	10	2	1	0,89
7 - Nutrição desequilibrada: menos que as necessidades corporais	0	0	10	2	1	0,89
8 - Risco de volume de líquidos desiquilibrados	0	0	10	2	1	0,89
9 - Risco de constipação	0	3	7	2	0,75	0,63
10 - Distúrbio no padrão de sono	0	1	9	2	0,92	0,84
11 - Risco de infecção	0	0	10	2	1	0,89
12 - Processo perinatológico ineficaz	0	0	10	2	1	0,89
13 - Dor aguda	0	0	10	2	1	0,89
14 - Baixa autoestima situacional	0	0	10	2	1	0,89
15 - Disposição para paternidade ou maternidade melhorada	0	0	10	2	1	0,89
16 - Processos familiares interrompidos	0	0	10	2	1	0,89
17 - Ansiedade	0	0	10	2	1	0,89
18 - Medo	0	0	10	2	1	0,89
19 - Risco de tensão do papel de cuidador	0	1	10	1	0,92	0,84
20 - Risco de vínculo prejudicado	0	0	10	2	1	0,89
21 - Conforto prejudicado	0	1	9	2	0,92	0,84
22 - Distúrbio na imagem corporal	0	0	10	2	1	0,89
23 - Disposição para bem-estar espiritual melhorado	0	1	9	2	0,92	0,84
24 - Sofrimento espiritual	0	1	9	2	0,92	0,89
Índice de Validade de Conteúdo total					0,96	0,86

*I - Inadequado; PA - Parcialmente adequado; A - Adequado; TA - Totalmente adequado; IVC - Índice de Validade de Conteúdo.*

Nesta etapa de validação, o único item que não atingiu o IVC para ser considerado validado foi a ligação N° 9 referente ao DE “Risco de constipação”, que teve o IVC de 0,75. Dessa forma, optou-se pela exclusão do item, considerando os apontamentos registrados pelos especialistas, que não consideraram esse DE relevante para o contexto de BLH.

## DISCUSSÃO

A atuação do profissional enfermeiro na promoção, proteção e apoio ao AM é realizada em diversos cenários, e para tal, o uso de tecnologias tem se destacado. Tecnologias educacionais, seguidas das assistenciais, são as mais empregadas na comunicação com mulheres em AM^(21)^. As tecnologias utilizadas no cuidado de enfermagem são resultantes da articulação de conhecimento científico para produção de bens materiais ou não materiais em prol de intervenções resolutivas na prática do cuidado^(22)^.

A assistência de enfermagem em BLH, guiada pela CE, promove autonomia, elevando a qualidade do cuidado prestado pelo enfermeiro^(23)^. A CE deve ser pautada nas etapas do PE, que, por sua vez, é reconhecido como uma estratégia que visa integrar as melhores práticas, fundamentando-se em evidências científicas, conhecimento clínico e necessidades do indivíduo^(24)^.

A construção de ferramentas específicas para coleta de dados, investigação, organização e análise de dados aprimora a eficiência e confiabilidade dos instrumentos^(18)^. Na enfermagem, a validação de ferramentas que orientam a prática representa um avanço nas tecnologias de saúde. Por meio desses instrumentos validados, é possível oferecer uma orientação mais eficaz no cuidado ao paciente, contribuindo para melhoria da qualidade da assistência prestada^(25)^.

O instrumento para implementação do PE em BLH atingiu o IVC mínimo recomendado na literatua^(17)^, o que reforça sua relevância. O instrumento desenvolvido para implementação do PE em BLH guia a CE, documentando a assistência de enfermagem às nutrizes.

Desenvolver e validar instrumentos para apoiar a implementação do PE em BLH corrobora a promoção, proteção e apoio ao AM, permitindo uma coleta de dados abrangente e organizada, facilitando a definição dos DEs e, por conseguinte, a realização das etapas subsequentes do PE.

Neste sentido, o uso de instrumentos para avaliação de enfermagem vem crescendo progressivamente na prática de enfermagem. Fundamentados nos princípios da Teoria das Necessidades Humanas Básicas, contribuem para assistência holística com foco na individualidade do paciente^(26)^.

A utilização de uma ferramenta que padroniza o cuidado confere assertividade na assistência de enfermagem. Esta foi a constatação de pesquisa em que foi construído e validado um instrumento para CE ao paciente pediátrico em pré-operatório fundamentado na Teoria das Necessidades Humanas Básicas, contemplando ligações NANDA-I, NIC e NOC, o qual atingiu IVCs de 0,90 e 1^(27)^.

A primeira parte do instrumento para implementação do PE em BLH, construído neste trabalho, contempla dados sociodemográficos, informações importantes para compreender o contexto em que ocorre no AM, pois fatores relacionados à idade, nível de escolaridade e situação socioeconômica influenciam a prática do AM. Em uma correlação entre renda materna e alimentação do recém-nascido (com leite materno + complemento em todas as alimentações), uma renda de três ou mais salários mínimos impactou negativamente para a prática do AM exclusivo na maternidade^(28)^.

Em contrapartida, a escolaridade materna se mostra determinante para o sucesso do AM. Dessa maneira, com a utilização de uma ferramenta que guie a avaliação de enfermagem, identificando as singularidades de cada contexto, o enfermeiro direciona suas intervenções por meio do seu julgamento clínico^(29)^.

Um dos apontamentos realizados pelos especialistas para inclusão de informações no instrumento construído foi referente à participação em atividades de educação perinatal e orientações referentes ao AM no decorrer do pré-natal. Essa sugestão vai ao encontro do protocolo 19 da *Academy of Breastfeeding Medicine* ^(30)^, revisado em 2024, que aborda a importância da promoção da amamentação no contexto pré-natal.

O protocolo enfatiza a importância da preparação das gestantes para amamentação, realizada por profissionais durante o pré-natal, abordando os benefícios do AM, desmitificando informações errôneas, promovendo e direcionando a gestante para grupos de apoio à amamentação, assim como o ensino de habilidades práticas como pega e posicionamento. O protocolo destaca a importância do envolvimento do parceiro/rede de apoio durante todo processo e realização de acompanhamento contínuo após nascimento^(30)^.

No entanto, estudo de coorte prospectivo com 300 puérperas constatou que a realização de orientações sobre AM no período gravídico-puerperal está aquém do recomendado, pois cerca de 50% da população estudada não recebeu orientação profissional sobre o AM^(31)^.

As informações dos blocos subsequentes do instrumento para avaliação de enfermagem sobre os antecedentes obstétricos, condições da amamentação atual e avaliação das mamas permitem identificar obstáculos e complicações com impacto direto na saúde da nutriz e desfecho do AM. No que diz respeito ao acompanhamento da gestação e ao número de consultas realizadas, observam-se diferenças estatisticamente relevantes na prevalência da amamentação aos seis meses. As gestantes que realizaram cinco ou mais consultas mantiveram a lactação aos seis meses, ao contrário das que realizaram menos de cinco consultas^(29)^.

Reforçando essa premissa, estudo sobre autoeficácia na amamentação identificou que as condições sociodemográficas, antecedentes obstétricos, gestação atual e puerpério impactam a autoeficácia. A nutriz deve receber acompanhamento especializado, e, de acordo com essa abordagem, é fundamental que os BLHs organizem a assistência por meio de ferramentas estruturadas para prestar assistência de qualidade^(32)^.

As condições mamárias, como produção insuficiente de leite materno, ingurgitamento, fissuras, fadiga, cansaço e dor, são informações importantes que impactam a saúde física e emocional das nutrizes, exigindo práticas adequadas para minimizar os impactos e promover a manutenção da lactação^(33)^.

Ao examinar fatores que influenciam a interrupção do AM exclusivo, os autores^(34)^ identificaram que a mesma ocorreu principalmente pela falta de conhecimento, assim como pelas crenças limitantes, interferência de amigos e familiares, evidenciando que não se pode considerar apenas os aspectos biológicos do AM, masconsiderar concomitantemente os fatores psicológicos e socioculturais.

Ao examinar fatores modificáveis em um mês pós-parto relacionados à amamentação exclusiva em três meses pós-parto, focando nas variáveis psicossociais, como complicações mamárias, autoeficácia na amamentação, níveis de estresse, sintomas depressivos, vínculo e apoio familiar, concluiu-se que tais fatores influenciam a continuidade da amamentação exclusiva^(34)^.

Dessa maneira, uma ferramenta que contemple a avaliação da nutriz na sua singularidade, considerando as condições psicobiológicas, sociais e espirituais, alicerçada na matriz NNN para definição dos DEs, resultados e intervenções, corrobora para identificação, planejamento e resolução precoce de problemas que podem impactar a manutenção da lactação.

### Limitações do estudo

Como limitação do presente estudo, consideraram-se as dificuldades em conseguir especialistas dispostos a participar do estudo e que atendessem aos critérios propostos. O fato de o estudo de ter sido conduzido com especialistas de um único país pode ter restringido a generalização dos resultados.

### Contribuições para as áreas da enfermagem, saúde ou políticas públicas

Utilizar um instrumento construído e validado com foco nas nutrizes não apenas guia e padroniza procedimentos, mas também aprimora a avaliação de enfermagem, o julgamento clínico do enfermeiro, o registro adequado das intervenções e os resultados alcançados. Seu potencial inovador corrobora para promoção da saúde materna e infantil no cenário onde for utilizada, podendo se estender a outros cenários, como consultórios de enfermagem, ambulatórios de amamentação, assim como para Atenção Primária à Saúde, sendo utilizado na CE.

## CONSIDERAÇÕES FINAIS

A pesquisa evidenciou a importância da construção de uma tecnologia para qualificação do cuidado às nutrizes. O PE, fundamentado nas taxonomias NANDA-I, NIC e NOC, permite a sistematização do cuidado, promovendo uma abordagem integral, individualizada e cientificamente embasada.

Com a construção da tecnologia assistencial, observamos que a enfermagem desempenha um papel central na identificação das necessidades das nutrizes, na proposição de intervenções práticas e na avaliação de resultados, contribuindo para desfechos favoráveis na manutenção da lactação.

A implementação do PE fomenta a autonomia profissional e padronização das práticas. No cenário dos BLHs, fortalece o compromisso do enfermeiro na promoção, proteção e apoio ao AM.

O IVC alcançado no presente estudo evidencia a confiabilidade do material desenvolvido como instrumento aplicável na prática profissional de enfermagem, especificamente no cenário do BLH.

## Data Availability

Os dados de pesquisa estão disponíveis no corpo do artigo.
